# A Look Inside HIV Resistance through Retroviral Protease Interaction Maps

**DOI:** 10.1371/journal.pcbi.0030048

**Published:** 2007-03-09

**Authors:** Aleksejs Kontijevskis, Peteris Prusis, Ramona Petrovska, Sviatlana Yahorava, Felikss Mutulis, Ilze Mutule, Jan Komorowski, Jarl E. S Wikberg

**Affiliations:** 1 Department of Pharmaceutical Biosciences, Uppsala University, Uppsala, Sweden; 2 Linnaeus Centre for Bioinformatics, Uppsala University, Uppsala, Sweden; The Scripps Research Institute, United States of America

## Abstract

Retroviruses affect a large number of species, from fish and birds to mammals and humans, with global socioeconomic negative impacts. Here the authors report and experimentally validate a novel approach for the analysis of the molecular networks that are involved in the recognition of substrates by retroviral proteases. Using multivariate analysis of the sequence-based physiochemical descriptions of 61 retroviral proteases comprising wild-type proteases, natural mutants, and drug-resistant forms of proteases from nine different viral species in relation to their ability to cleave 299 substrates, the authors mapped the physicochemical properties and cross-dependencies of the amino acids of the proteases and their substrates, which revealed a complex molecular interaction network of substrate recognition and cleavage. The approach allowed a detailed analysis of the molecular–chemical mechanisms involved in substrate cleavage by retroviral proteases.

## Author Summary

Retroviruses are associated with a broad range of diseases that include tumor formation, neurological disorders, and immunodeficiency syndromes, including those of HIV. The extraordinary mutational plasticity of HIV-1 causes the rapid appearance of highly diverse quasi-species in a very short time, leading to severe problems with drug resistance. We here present and validate experimentally a novel approach for the analysis of the molecular interaction networks involved in the recognition process of substrates by natural and drug-resistant retroviral proteases. By combining a large number of wild-type and mutant retroviral proteases from nine different viral species, and their interactions with a large number of substrates, we have created a unified model incorporating all the proteases' mutational space. Our results reveal that a complex physicochemical interaction network is involved in substrate recognition and cleavage by aspartate proteases and unravel detailed molecular mechanisms involved in drug resistance. These findings provide novel implications for understanding important features of HIV resistance and raise the possibility of developing completely novel strategies for the design of protease inhibitors that will remain effective over time despite rapid viral evolution.

## Introduction

Retroviruses are associated with a broad range of diseases that include tumors, immunodeficiency syndromes, and neurological disorders [1]. They affect a large number of species, from fish and birds to mammals and humans, with global socioeconomic negative impacts [1]. Each year the HIV pandemic causes more than 3 million deaths despite advances in the development of anti-HIV therapies [2]. The seemingly endless capability of retroviruses to escape antiviral drugs undermines treatment strategies and prompts the need for new broad-spectrum therapeutic agents [3].

Retroviral proteases process viral precursor polyproteins into structurally and functionally mature proteins that combine into infectious viral forms. As such, these proteins are key targets for the design of therapeutic inhibitors [4,5]. To date, the majority of protease inhibitors for treatment of HIV have been peptide mimetics, and most of them were specifically designed against only one of the HIV-1 proteases, namely the HXB2 (“wild-type”) HIV-1 protease [6,7]. Unfortunately, this strategy has led to failures to retard the replication of strains bearing drug-resistant protease mutations [3,8].

Although efficiently hydrolysable protease substrates have served as excellent templates for peptide-mimetic inhibitor design, it is difficult to predict which combination of amino acids will make the best substrate over multiple proteases [6]. Analysis of protease mutations associated with drug resistance is also confounded by the existence of many viral subtypes carrying naturally occurring polymorphisms [9]. The genomic differences among HIV-1 proteases can be as high as 30% and range from 10%–70% within the retroviral protease class [3]. Mutations contributing to viral resistance to antiviral drugs in one particular HIV subtype are found frequently in equivalent positions in the genes of other HIV subtypes or other retroviral proteases [9–14]. Still, the roles of specific mutations are only partly understood [5].

Here we report the development and experimental validation of a novel strategy for the molecular analysis of retroviral proteases. We hypothesized that merging essentially all available knowledge of retroviral proteases and their interactions with their substrates into a unified model would provide broad insight into the function of these enzymes and facilitate the analysis of retroviral drug resistance mechanisms. The modeling that we here report is based on the multivariate analysis of sequence position–physicochemical properties of the amino acids of 61 retroviral proteases from nine viral species and reveals a complex network of physicochemical interactions involved in protease recognition and cleavage of substrates. The approach provides novel insights into the molecular mechanisms involved in substrate cleavage by retroviral proteases in general as well as in relation to drug resistance.

## Results

### Substrate CRM for Retroviral Proteases

The model was based on an extensive survey of publicly available data from multiple retroviral proteases and their substrates from 16 years of retrovirus research during 1990 to 2005, combined into a single dataset (Table S1). Because retroviral proteases are inherently dynamic structures that undergo significant structural changes with binding, we described each structurally aligned amino acid of the 61 retroviral proteases by their principal physicochemical properties (i.e., their z-scales z_1_–z_5_), rather than using the proteins' static 3-D structures (see Materials and Methods, Figure S1, and Table S2) [15,16]. Similarly, we described the retroviral protease substrates by considering the same principal physicochemical properties of every single amino acid of the octapeptide sequence spanning the P_4_ to P_4_′ position (see Materials and Methods for details).

Protease cleavage rates are dependent on the constituents of the experimental assay (e.g., pH and salt concentrations) [17]. To account for differences in the assays, additional assay descriptors were introduced (Table S3). Substrate recognition and cleavage involve many dynamic noncovalent and covalent interactions between the substrate and the enzyme. Such complex processes can be accounted for by introducing “cross-terms” into the multivariate modeling. Cross-terms are formed as a product of multiplication of any two of the descriptors and reflect the simultaneous influence of two particular physicochemical properties on activity. Cross-terms can be viewed as description of specific interactions, which do not necessarily need to occur by physical contact of amino acids with each other. In a mathematical sense, cross-terms represent approximate nonlinear contributions of combination effects, regardless of whether these occur due to close contact or not [18,19].

**Figure 1:**
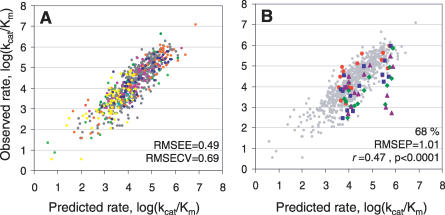
Goodness of Fit and Experimental Validation for the CRM (A) Observed versus predicted rate of cleavage of 299 substrates by 61 retroviral proteases (in total, 760 protease–substrate combinations) for the CRM, in which all predictions relate to model-building data (R^2^ = 0.77; Q^2^ = 0.52). Each bullet represents naturally occurring and artificially mutated protease forms of HIV-1 (gray), HIV-2 (magenta), AMV (light green), RSV (blue), HTLV-1 (orange), BLV (red), Mo-MuLV (yellow), EIAV (green), and FIV (light blue). (B) A priori prediction of cleavage rates of 15 novel peptides with diverse structures by the CRM (Table 3, numbers 4–18). Shown is the predicted versus experimentally determined cleavage rates by HXB2 (red) and mutant HIV-1 proteases, I84V (blue), L90M (magenta), and I84 + L90M (green). The prediction error for the cleavage rates was less than one log(k_cat_/K_m_) unit for 68% of the protease–substrate pairs; the correlation for the a priori predicted rates versus the experimentally determined rates yielding a correlation coefficient *r* = 0.47 (*p* < 0.0001), as indicated on the panel. The data in (A) is also shown in (B) (gray).

The descriptors of the retroviral proteases, substrates, assays, and cross-terms were correlated to the experimentally determined substrate cleavage rates (k_cat_/K_m_) using partial least squares (PLS) regression modeling (Table 1). Our results show that it is possible to obtain acceptable models only after inclusion of cross-terms between the descriptors of amino acids in the substrates and proteases, and between amino acids at different positions in the substrates (Table 1). Moreover, including assay descriptors in the modeling further increased the validity of the model (Table 1). The performance of the cleavage rate model (CRM) is summarized in Table 2 and shown graphically in Figure 1A.

**Table 1:**
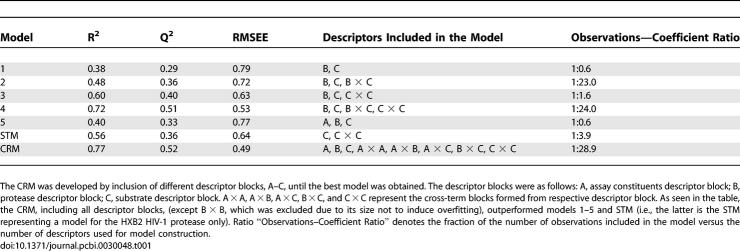
Creation of a Substratethe CRM for Retroviral Proteases

**Table 2:**
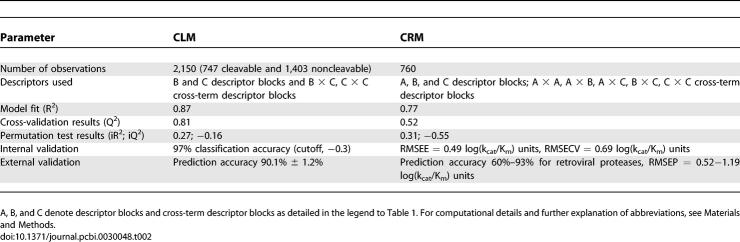
Details of the CLM and CRM Obtained Herein, and Their Validation Results

**Table 3:**
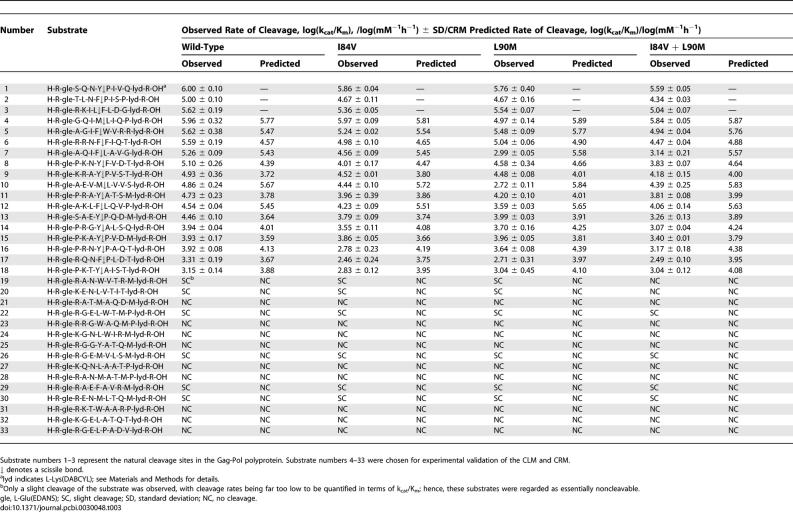
Experimentally Determined Cleavage Rates of 33 Peptide Sequences by HXB2 and Mutant HIV-1 Proteases

### External Validation of CRM

**Figure 2:**
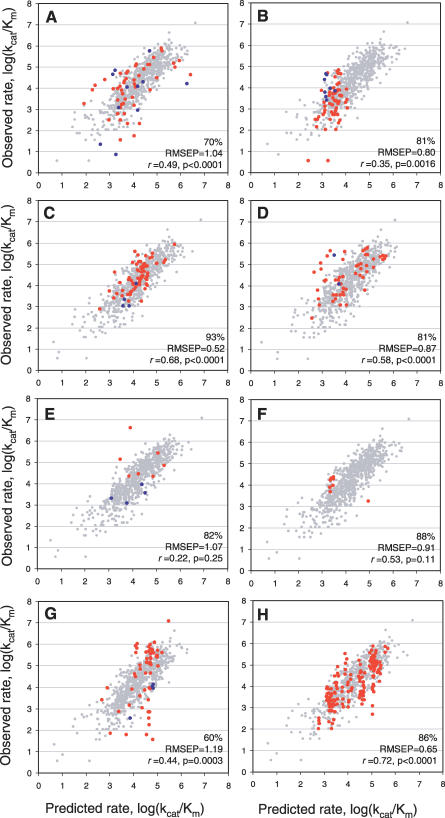
External Predictions for Retroviral Proteases by CRMs Each panel represents the predictions of a model created on the data collected herein (Table S1), but excluded all data for the proteases of one retroviral strain, one at a time, and uses the model to predict the excluded data. Blue bullets correspond to prediction of cleavage activity for new proteases and new substrates (i.e., the cases in which neither the protease nor the peptide was represented in the dataset used in creation of the model). Red bullets correspond to prediction of the cleavage rates for the new proteases only (i.e., the cases in which the peptide, but not the protease, was represented in the dataset used for model creation). Gray dots represent the observed versus computed cleavage rates for each of the respective models (i.e., the data used for model creation). (A–H) represent external predictions for wild-type, naturally occurring, and artificially mutated proteases as follows: (A) AMV, (B) Mo-MuLV, (C) HIV-2, (D) RSV, (E) EIAV, (F) FIV, (G) HTLV-1, and (H) HIV-1 proteases. (For [H] the data excluded were for 23 mutant HIV-1 proteases, with mutations associated with drug resistance.) The percentages in each panel indicate the fraction of predicted observations with a prediction error less than one log(k_cat_/K_m_) unit. RMSEP, the correlation coefficients (showing correlation between the observed versus the externally predicted rates), and their significances are shown on each panel.

To validate the model further we examined its capacity to predict the activity of naturally occurring and artificially mutated retroviral proteases externally. This was afforded by excluding all data for eight retroviral strains one at a time in their entirety, and then predicting the excluded data using models constructed from the remaining data (see Materials and Methods for details). This analysis showed that the models could accurately predict the activities of the excluded retroviral proteases, most notably for the HIV-2 protease with an accuracy of 93% (root mean square error of prediction [RMSEP] = 0.52), and for the HIV-1 protease mutants 86% (RMSEP = 0.65; Figure 2). By contrast, state-of-the-art model building using only the HXB2 HIV-1 protease data failed to give acceptable models (Table 1; see Materials and Methods for further details).

### Experimental Validation of CRM

Our approach thus resulted in a statistically well-validated model for the rate of cleavage of peptide substrates by retroviral proteases. However, although the model was capable of predicting k_cat_/K_m_ values, it could not distinguish cleavable from noncleavable sequences. To allow such predictions, we constructed a cleavability model (CLM) by correlating substrate and protease descriptors and their cross-terms to a vector representing cleavability or noncleavability (Table 2; see Materials and Methods for details). The CLM, which was based on the data for all 61 proteases with all cleavable peptides used for the construction of the CRM as well as an additional large set of noncleavable peptides, almost perfectly classified cleavable and noncleavable substrates (97%) and performed excellently in external predictions of cleavability of new sequences (90.1% ± 1.2%; see Table 2 and Materials and Methods).

Encouraged by these results, we confirmed the predictive power of the models by independent experimental validation. We constructed a virtual peptide library and applied in silico screening to it, first by using the CLM, then followed by the CRM. This process resulted in an unbiased set of 30 novel peptides, selected according to diversity criteria, of which 15 were predicted as cleavable and 15 as noncleavable; the predicted cleavage rates for the cleavable ones ranging over almost three orders of magnitude (see Materials and Methods for details).

The peptides were subsequently synthesized and assayed for their cleavability by the HXB2 HIV-1 protease and three HIV-1 proteases harboring mutations associated with drug resistance. The analysis showed that the CLM could correctly recognize all cleavable substrates as cleavable, and all noncleavable substrates as essentially noncleavable (100% accuracy; Table 3). Moreover, the experimentally determined cleavage rates of the cleavable peptides agreed well with the CRM predicted rates on HXB2 and mutated HIV-1 proteases (68% accuracy; RMSEP = 1.01; Figure 1B). Addition of all experimental data to the CRM further increased CRM predictability according to cross-validation; the Q^2^ increased from 0.52 to 0.54.

### Interpretation of the Chemical Effects in Substrates Determining their Cleavage Susceptibility by Retroviral Aspartate Proteases

**Figure 3:**
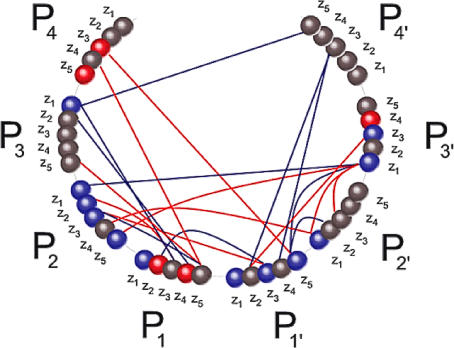
Map of Physicochemical Properties of Retroviral Protease Substrates Based on the Regression Coefficients of Substrate and Substrate–Substrate Cross-Terms of the CRM The figure summarizes the physicochemical requirements that a substrate should possess to be efficiently cleaved by a “swarm” of viral variants, notwithstanding that individual variants may favor or disfavor particular substrates. Spheres correspond to the five principal properties (z_1_–z_5_) for each amino acid [16]. Red spheres denote that a position favors an amino acid with a positive value of its z-scale for high cleavage rate. Blue spheres indicate that an amino acid with negative value of the z-scale is favored. For example, Ala, Asn, Asp, Pro, and Ser have positive values for z_3_ and z_5_ and are thus amino acids preferred for the P_4_ position for affording a substrate with higher cleavage activity. Lines indicate the most important substrate–substrate cross-terms. Red lines denote that when the z-scales of both amino acids show large positive or large negative values, higher cleavage activity is favored. Blue lines indicate that higher cleavage rates are favored when both the z-scales have large values with different tokens. Black spheres denote the remaining substrate amino acid properties, which have a smaller effect on efficient substrate cleavage (see Materials and Methods).

Analysis of the regression coefficients of the CRM allows analysis of the physicochemical properties of the amino acid residues of both the substrates and the proteases required for catalytic activity. The model verifies the well-known hydrophobic requirement for the P_3_–P_3_′ residues of the substrates by the retroviral protease cleavage sites (i.e., the regression coefficients for the z_1_ terms of the P_3_–P_3_′ residues of the substrates are negative, as can be seen from the physicochemical property map derived from the model; Figure 3) [20]. In fact, the map shows that P_3_ and P_3_′ can accommodate various amino acids with a preference for hydrophobic residues, which is in perfect agreement with previous findings [13]. Moreover, it is well-known that β-branched or polar amino acids are not tolerated in the P_1_ position of retroviral substrates [13]. In the model, this limitation is reflected in that the z_1_, z_2_, and z_4_ terms for the P_1_ position are most favorable for aromatic amino acids and methionine, while polar or β-branched residues are disfavored.

Specificity studies for retroviral proteases have found that highly complicated and not easily interpretable interior interactions take place in the substrates [21]. Such interactions become easy to interpret in the model by the cross-terms, however. For example, the large regression coefficients from particular physicochemical properties of the P_1_′–P_2_, P_1_′–P_1_, P_1_′–P_3_′, and P_1_–P_3_ residue pairs indicate the presence of interactions for these pairs, while no such interactions take place for the P_3_–P_2_ pair according to the model, in accordance with experimental results [22]. Cooperativity between P_1_–P_2_, P_1_–P_3_, and P_1_–P_4_ residue pairs, indicated by the model, has also been shown to be important for specificity features [21]. In earlier specificity studies of retroviral proteases, many series of substrates were used, each of which often differ only by one or two residues, prohibiting a complete analysis of all residues at every subsite [21]. Merging all the available data thus provides a comprehensive picture that reveals important cross-dependencies between several different residue positions in the substrates (i.e., the regression coefficients where particular substrate–substrate cross-terms are significantly large), and demonstrates that a complex interaction network between residues in the substrates is involved in their cleavage (Figure 3).

### Interpretation of the Chemical Effects in Aspartate Proteases Involved in their Cleavage of Substrates

In a similar way as above for analysis of substrates, the regression coefficients of the descriptor terms for protease amino acid residues can identify the physicochemical properties of the nonconserved amino acids in the proteases that determine substrate cleavage (e.g., the model reveals that hydrophobic amino acids are preferred at the position corresponding to position 82 in HIV-1 protease to afford a high catalytic activity of the proteases). This is due to the fact that the regression coefficient z_1_ for position 82 is the largest one and negative. Another example is that hydrophobic, small-size amino acid residues (e.g., Ile or Pro) are preferred at position 81, since both regression coefficients for hydrophobicity (z_1_) and size (z_2_) are among the largest and also negative at this position for the model.

**Figure 4:**
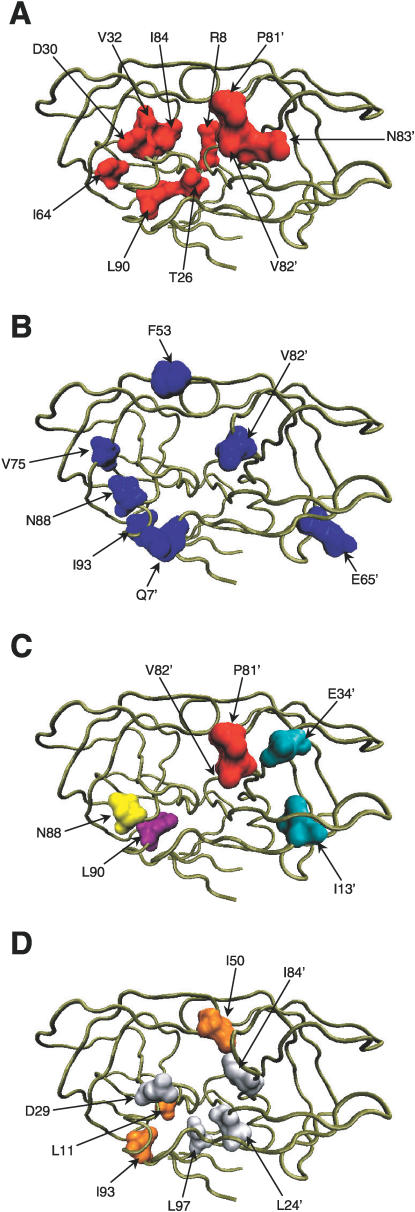
The Ten Most Important Nonconserved Residues in Retroviral Protease for Substrate Recognition and the Most Important Cross-Dependences of Retroviral Protease and Substrate Amino Acids Identified by Use of the CRM (A) The amino acids are shown in red on the 3-D structure of the HXB2 HIV-1 protease as a template. Because the retroviral proteases are homodimers, the modeling does not allow a distinction between cases where only one or both of the amino acids of the homoprotein should be assigned as important. (B) The retroviral protease amino acid residues most important for P_4_ substrate position (shown in blue). (C) The retroviral protease amino acid residues most important for the P_3_ (light blue), P_2_ (yellow), P_1_ (red), and P_1_′ (magenta) substrate positions. (D) The retroviral protease amino acid residues most important for the P_3_′ (white) and P_4_′ (orange) substrate positions (see Materials and Methods for details).

To assess a cumulative importance of all physicochemical properties for each protease residue relatively to other residues, we computed and compared the absolute value sums of z_1_–z_5_ regression coefficients for each individual position, which is thus a measure representing the overall importance of an amino acid in eliciting chemical effects in the protease when compared to the same measure of other amino acids in the model. Our results from this analysis reveal the most important nonconserved positions involved in catalytic activity of the retroviral proteases (Figure 4A; see Materials and Methods for details). One of the most important amino acid residues shown by the CRM was the threonine of the aspartate proteases' catalytic triad, Asp-Thr(Ser)-Gly (i.e., the T26 residue in HIV-1 protease that is substituted to serine in Rous sarcoma and avian myeloblastosis virus retroviral proteases; Figure S1) [23]. Six further amino acid positions (corresponding to R8, D30, V32, V82, I84, and L90 residues in the HXB2 HIV-1 protease) were also identified as important. These positions agree well with the amino acids known to be associated with high resistance to protease inhibitors [24,25]. The model also identified P81 and N83 amino acid positions, which are known to play a key role in regulation of retroviral protease function [26]. The role of the I64 residue, also indicated by the model, appears to be indirect, as it is located farther way from the substrate cleavage cleft (Figure 4A).

The substrate-protease cross-terms of the CRM were then in a similar fashion used to identify the major cross-dependencies of the protease and substrate amino acids for cleavage activity, which thus reveal the major interaction effects that determine substrate specificity (Figure 4B–4D; see Materials and Methods for details). We then found that P_3_′ substrate residues form the strongest cross-dependencies with retroviral protease amino acids corresponding to L24, D29, I84, and L97 residues in the HIV-1 protease (Figure 4D). Notwithstanding that D29 directly contributes to the S_3_′ subsite*,* the effect of residues L24, I84, and L97 distal to the S_3_′ subsite is indirect [13]. Further analysis indicated the importance of direct interactions between the P_1_ residue and the P81 and V82 protease amino acids (Figure 4C), which form a part of the S_1_ subsite [13].

The P_1_′ residue, on the other hand, shows a major indirect interaction with the L90 amino acid (Figure 4C). This is a position for a distantly acting, commonly appearing drug-resistant mutation, L90M in the HIV-1 protease, which has been observed to increase the cleavage activity of HIV-1 protease for natural substrates mutated in the P_1_′ position [27]. Although the P_3_ amino acid may interact directly with various amino acids in the S_3_ pocket, our results suggest that the P_3_ amino acid specificity is determined indirectly by effects arising from the I13 and E34 residues (Figure 4C). This result is in alignment with other reports, where the polymorphic mutation I13V was linked with the mutation of Thr to Ala at the P_3_ position of the natural cleavage site p24/p2 [28]. Moreover, mutations at the E34 position have been seen in clinical HIV samples after protease inhibitor treatment [29].

The analysis further demonstrated that the P_4_ and P_4_′ residues form a large number of important cross-terms with protease amino acids (Figure 4B–4D). The P_4_ and P_4_′ positions can broadly tolerate a variety of amino acids (Figure 3). However, mutations could occur in the P_4_ and P_4_′ positions of natural cleavage sites under antiviral drug pressure, which compensate a decreased catalytic activity of drug-resistant retroviral multi-mutants with the mutations depicted in Figure 4B–4D. Indeed, resistance mutations are known at such positions. For example, the Cbz group of the retroviral protease inhibitor TL-3 occupies the S_4_ subsite and interacts with the F53 residue, where mutation to a smaller Leu causes a decreased susceptibility of TL-3 by an order of magnitude [30]. Moreover, a substantial overlap exists between retroviral protease residues associated with specificity (Figure 4B–4D) and residues involved in resistance development (I13, I50, F53, V82, I84, N88, L90, and I93) [24].

## Discussion

A goal of any successful antiretroviral therapy must be to ensure complete inhibition, or at least a fair retardation, of the replication of all the multiple viral strains that constantly emerge in an infected organism. The present approach allows concomitant analysis of many mutated target proteases in silico, and is useful to aid the analysis of the roles of such mutations in drug resistance. The Stanford HIV drug resistance database contains more than 24,000 HIV protease polymorphisms and resistance mutation sequences [31]. Performing high-throughput screenings and ligand optimizations to search for a drug suited to such a multitude of targets is an insurmountable task. Traditional structure-based drug design is built on the “lock-and-key principle,” in which a drug is designed to be a snug fit with its target protein [3]. It is not well-suited to concomitant design of multiple targets that undergo conformational changes and show dynamically regulated differences in the shape of their active sites. Our results show that combining multiple proteases from many retroviral strains encompasses the mutational space information of retroviral proteases better than using the protease from a single strain. Thereby, it becomes possible to obtain models that allow interpretations of the molecular mechanisms involved in retroviral protease cleavage site processing. The multiple-protease–based models thus allow localization of physicochemical effects that rule substrate cleavage and predict multiple positions where compensational mutations could occur that restore substrate cleavage following the appearance of protease inhibitor resistance mutations. The validity of the models are proven not only by applying state-of-the-art statistical validation methods, but also by their ability to a priori accurately predict the cleavage rate of entirely novel peptides and proteases. Interestingly, the model also reveals several amino acids outside of the enzymes' binding pocket, such as I13, L24, E34, I64, I84, L90, and L97, as being important for catalytic activity. It is well-known that retroviral proteases are flexible proteins, and it is likely that these positions contribute with long-range conformational effects that indirectly affect protein function and mobility [18].

The regression coefficients of terms and cross-terms of the model contain a large amount of chemical information that would be of direct value in designing a substrate that is efficiently cleaved over a group of protease mutants. Another option for such design would be to apply virtual screening of peptide libraries using the model. In addition, we show that inclusion of new experimental data leads to a model with improved predictability. Iterating the process should thus give models that afford increasingly accurate predictions of peptides with particular properties (e.g., having broad specificity over multiple resistance mutations). Analyzing such new entities experimentally and including the new data into new models would lead to further improved models and would refine the understanding of how retroviral proteases overcome drug resistance.

## Materials and Methods

### Data and data preprocessing.

Data for substrate cleavage by 61 retroviral proteases were collected in an extensive survey that included publicly available data for retroviral proteases during 1990–2005 [32–64]. The survey included proteases from the following viruses: HIV-1, HIV-2, AMV (avian myeloblastosis virus), RSV (Rous sarcoma virus), HTLV-1 (human T cell leukemia virus type 1), BLV (bovine leukemia virus), Mo-MuLV (Moloney murine leukemia virus), EIAV (equine infectious anemia virus), and FIV (feline immunodeficiency virus); its outcome is summarized in Tables S1 and S2. In some cases fully denaturated proteins had been exposed to HIV-1 or HIV-2 proteases [61,62,64]. Noncleavable octapeptides were in these cases extracted from the noncleavable fragments located between the observed cleavage sites by using an eight-residue-long sliding window. Some of the data were generated in-house as described below (see Materials and Methods further below).

*Description of proteases.* The 61 retroviral protease sequences included in the study (Table S2) were aligned using the template shown in Figure S1. A total of 94 amino acids could be fully aligned over all the proteases, but only the positions lacking gaps in all proteases, as well as those being nonconserved, were considered. These then amounted to 85 amino acids, which were described by their five principal physicochemical properties, or “z-scales” [16]. These z-scales roughly represent hydrophobicity (z_1_), steric properties (z_2_), polarizability (z_3_), and polarity and electronic effects of amino acids (z_4_, z_5_) (z-scales are the principal components of 26 physicochemical properties of amino acids, which include: molecular weight, van der Waals volume, heat of formation, energy of highest occupied molecular orbital, energy of lowest unoccupied molecular orbital, log P, α-polarizability, absolute electro-negativity, absolute hardness, total molecular surface area, polar molecular surface area, nonpolar molecular surface area, number of hydrogen bond donors, number of hydrogen bond acceptors, indicator of positive charge in the side chain, indicator of negative charge in the side chain, NMR α-proton shifts at pD = 2.7 and 12.5, and seven descriptors representing thin-layer chromatographic mobilities using different stationary and mobile phases [16]).

Thus, every protease was described by 85 × 5 = 425 descriptors, which comprised the physicochemical property space information of the series of proteases used herein. It shall be noted that amino acids entirely conserved in a library do not yield any additional information and their importance can therefore not be assessed unless the library is extended by further mutations of these positions.

*Description of substrates.* We restricted the length of the substrates to octapeptides (P_4_-P_3_-P_2_-P_1_-P_1_′-P_2_′-P_3_′-P_4_′, where P_4_ represents substrate N-terminus amino acid and P_4_′ represents C-terminus substrate amino acid), since generally only eight amino acid residues are involved in the interaction process with eight subsites (S_4_-S_3_-S_2_-S_1_-S_1_′-S_2_′-S_3_′-S_4_′) of a retroviral protease, with the cleavage site being between the P_1_ and P_1_′ amino acids. Each one of the eight amino acids of the substrates were described by the same five z-scales as above, yielding 8 × 5 = 40 total descriptors for each substrate. This comprised the physicochemical space information of the series of substrates used herein.

*Description of assay conditions.* Descriptors for eight constituents of the experimental assays according to the published data used [32–64] were included in the modeling in order to normalize for the differences in assay conditions. The descriptors used are given in Table S3 and accounted for variations in pH, sodium chloride, 2-mercaptoethanol, EDTA, DMSO, dithiothreitol, nonidet-P40, and glycerol concentrations.

*Description of cross-dependencies of proteases, substrates, and assays.* The mutual dependencies of protease, substrate, and assay properties were described by cross-terms. These cross-terms were formed by multiplication of any two of the above-described descriptors of proteases, substrates, and assays. To simplify the discussion in the following, the above blocks of descriptors for assays, proteases, and substrates will be referred to as A, B, and C descriptor blocks, respectively. The cross-terms were then formed by multiplications yielding A × A, A × B, A × C, B × C, and C × C cross-term blocks. Each one of these blocks were in the subsequent modeling used in various combinations, together with the A, B, and C blocks, to demonstrate their respective importance and to find the most suited combination for creation of optimal models. All ordinary protease, substrate, and assay descriptors were mean-centered and scaled to unit variance prior to computation of cross-terms. In addition, we applied block-scaling for each type of descriptors to account for their differences in number and mutual correlation [65]. (Block-scaling gives each block a variance square root of N_b_, where *N* is the number of descriptors in block *b*. Block-scaling thus gives each variable the variance 1/(N_b_)^1/2^. The procedure avoids a situation where large blocks of descriptors mask small ones.) (The B × B cross-terms block was not formed due to its huge number of descriptors [i.e., 90,100 descriptors]).

### Description of the kinetics of experimental data.

Two types of models were created. One aimed to delineate whether or not a peptide is cleavable by retroviral proteases. This model, called CLM, was trained against a vector formed by assigning +1 to a hydrolysable substrate and −1 to a nonhydrolysable. The other model aimed to model the cleavage rate of cleavable substrate, and was called CRM. In the latter case, the model was trained against the vector formed from the logarithm of the experimentally determined k_cat_/K_m_ values (mM^−1^h^−1^ units), log(k_cat_/K_m_).

### Multivariate modeling and data analysis.

*CRM.* All experiments listed in Table S1, where substrate cleavage rates had been determined, were used for the construction of CRM, and comprised 760 observations. Protease, substrate, and assay descriptors, and cross-terms thereof, were used as detailed in Table 2. The preprocessed descriptors (see below) were correlated to measured cleavage rates log(k_cat_/K_m_) units by PLS regression modeling using Simca-P+ 10.0 software (Umetrics AB, http://www.umetric.com). In the model building, inclusion of various descriptor blocks were attempted and the data were subjected to PLS regression modeling (see models 1–5 and the single target model [STM] in Table 1 for details) [65]. While models 1–5 utilized all the 760 log(k_cat_/K_m_) values obtained from Table S1, the STM comprised only 212 experiments for the HXB2 HIV-1 protease of Table S1. Models were subjected to validation (see below), and model 4 and the CRM were the only ones considered acceptable (R^2^ > 0.7 and Q^2^ > 0.4) [66]. As CRM also outperformed model 4, it was the one used herein.

For the CRM containing descriptors of substrates, proteases, assays, and their cross-terms as shown in Table 2, the regression equation can be expressed as follows:

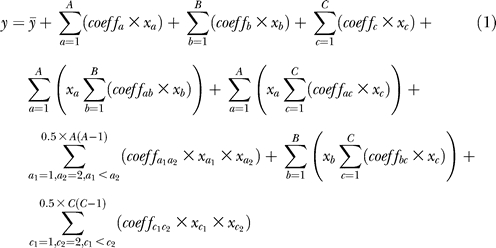
where *A*, *B*, and *C* represent the number of descriptors in assay, substrate, and protease blocks respectively, *a*, *b*, and *c* correspond to assay, substrate, and protease descriptors respectively, and *coeff* denotes a coefficient for a corresponding descriptor or a cross-term.

*CLM.* All data listed in Table S1 were considered for the CLM. Assay descriptors were not included. This was because the assay conditions used have only minor effects on substrate cleavability. In some cases, the assay conditions also had not been specified. All in all, the dataset comprised 2,163 peptide–protease combinations. However, 13 experiments of these differed only by assay descriptors and were therefore excluded. This resulted in a final dataset with a total of 2,150 observations, which was used for the model creation. Proteases, substrates descriptors, and cross-terms were used for the CLM construction as denoted in Table 2. The descriptors, preprocessed as described below, were correlated to the peptide cleavability (+1/−1) by PLS regression modeling using Simca-P+ [65].

For the CLM containing descriptors of substrates, proteases, and their cross-terms as shown in Table 2, the regression equation can be expressed as follows:

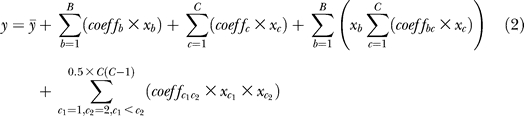
where *B* and *C* represent the number of descriptors in substrate and protease blocks, respectively, *b* and *c* correspond to substrate and protease descriptors, respectively, and *coeff* denotes a regression coefficient for a corresponding descriptor or a cross-term.

*Validation of models.* The goodness-of-model fits were quantified by R^2^. This unitless fraction indicates the portion of the total variation of the response that is explained by the model and shows how well a model fits the data [65,66]. We also computed the root mean square error of estimation (RMSEE) to determine the internal calculation error within the model:

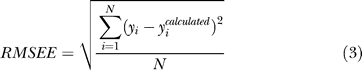
where *y_i_* and *y_ i_^calculated^* denote the observed and calculated rates by the CRM [65]. *N* denotes the number of calculated observations.

Cross-validation is a method of estimating the accuracy of a regression model. In cross-validation the dataset is divided into several parts (seven were used herein), with each part used to test a model fitted to the remaining parts, resulting in the cross-validated regression coefficient Q^2^ [67,68], where a higher Q^2^ denotes a better predictability [66].

In bootstrap validation the dataset is repeatedly and randomly permutated, yielding new dataset samples with replacements from the original dataset [69,70]. New models are then built on permutated data, and R^2^, Q^2^, and correlation coefficients between original and permutated response values are estimated. Intercept values for R^2^ (iR^2^) and Q^2^ (iQ^2^) reflecting R^2^ and Q^2^ of random response data were computed from repeated random permutations of the data (100 repeats were done herein) [70]. Negative iQ^2^ indicates that it is impossible to get predictive models based on random data.

External validation for the CLM was performed by randomly dividing the dataset into two parts (30% and 70%). The smaller part was excluded and predicted based on a model created from the remaining 70% of the data. This procedure was repeated ten times. For each external validation round we calculated the prediction accuracy (i.e., the fraction of correctly classified substrates to cleavable or noncleavable versus all observations included in the test set).

External validation of the CRM was performed by excluding all data for eight retroviral strains one at a time in their entirety, and then predicting the excluded data using models constructed from the remaining data. (In the case of HIV-1 proteases, the HXB2 HIV-1 protease and HIV-1 proteases with five artificial stabilizing mutations, Q7K + L33I + L63I + C67A + C95A, were kept in the model, and the external predictions were performed for the remaining 23 drug-resistant HIV-1 mutants.) The prediction accuracy for each model was estimated as the fraction of protease–substrate pairs with prediction error < 1.0 log(k_cat_/K_m_) to all protease–substrate pairs used for the respective external prediction. This critical threshold was set based on 2-fold RMSEE for the CRM (0.49 log(k_cat_/K_m_); Table 1). We also used RMSEP to evaluate model predictive ability for external datasets [65]. RMSEP can be compared with the root mean square error of internal cross-validation (RMSECV), which illustrates the error of predictions within the model [65]. RMSEP was computed as follows:

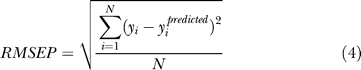
where *y_i_* denotes the observed rate and *y_ i_^predicted^* the externally predicted rate by the CRM. RMSECV was calculated in an identical fashion, using for *y_ i_^predicted^* the predicted rates obtained during internal cross-validation of the CRM [65]. *N* denotes the number of predicted observations.

The correlation coefficient, *r*, for the experimentally observed versus predicted cleavage rates by the CRM (Figure 1B and Figure 2A–2H) was determined, and the statistical significance, *p*, of the correlation was assessed. The *p*-value obtained is the probability that a correlation this great or greater (in the positive direction only) would be seen if there was no linear relationship between observed and predicted cleavage rates. An in-house add-in to Excel (Microsoft, http://www.microsoft.com) was used for the test of correlation. All significance tests were one-sided.

*Analysis of CRM.* All descriptors used for the CRM construction were mean-centered and scaled to unit variance, as described above. This transformation unified the different ranges of descriptor values allowing a comparative analysis of their coefficients. The larger an absolute value is of a descriptor's coefficient, the larger its impact is on the model's outcome.

To construct the retroviral protease substrate physicochemical fingerprint map shown in Figure 3, we analyzed CRM substrate and substrate–substrate cross-term descriptor coefficients. First, we compared the absolute values of the coefficients to find the largest ones. A total of 17 z-scales of the substrates' amino acid residues were then identified to be highly important and are shown in Figure 3 as a red sphere if its regression coefficient had a positive value, and as a blue sphere if it was negative. In a similar way we identified 20 highly important substrate–substrate cross-terms. These are represented in Figure 3 as red lines if the corresponding cross-term coefficient had a positive value, and as blue lines if it was negative.

To determine the most important protease amino acids shown in Figure 4A, we compared the sum of the absolute values of the five z-scale descriptor coefficients for each of the 85 aligned amino acid positions of the proteases. Summation of the coefficients allowed us to simultaneously capture all the physicochemical property effects caused by each of the amino acids considered. The ten amino acids with the largest sums of their coefficients and consequently the largest contribution on cleavage rate according to the model were the ones depicted in Figure 4A. Figure 4A was produced using the Visual Molecular Dynamics (VMD) program, version 1.8.3 [71].

The model was further analyzed by considering protease–substrate amino acid interactions as described by cross-terms. Every protease–substrate amino acid pair yields 25 cross-terms, as five z-scales of each amino acid multiplied makes 25 cross-terms. To capture the most important protease–substrate interactions, we calculated the sum of the absolute values of 25 cross-term coefficients for each substrate–protease amino acid pair. We then compared all obtained sums and identified the protease–substrate interactions with the largest influence on the model's outcome. In total, the 20 most important protease–substrate amino acid pairs are presented in Figure 4B–4D. Figure 4B–4D was also produced using VMD [71].

It may be noted that whereas the regression coefficients arising from the substrates only, or the proteases only, relate to the overall activity of all the substrates and all the proteases, respectively, the coefficients of the substrate–protease cross-terms relate to specificity (i.e., the ability of a particular substrate to prefer a particular protease).

*In silico substrate screening.* The active site of HIV-1 protease accommodates a sequence of eight amino acid residues (P_4_–P_4_′) of a substrate, and cleaves it between the P_1_ and P_1_′ residues. The potential number of substrates consisting of natural amino acids is therefore 20^8^, but this large number was not computationally feasible to assess. We therefore constructed a smaller library of octapeptide sequences by considering only the natural amino acids that can frequently be found in retroviral protease substrates as follows: for the P_4_ position, amino acids were R, S, K, P, G, or A; for P_3_, Q, A, R, K, G, or E; for P_2_, N, E, A, G, T, I, L, or V; for P_1_, F, Y, W, M, or L; for P_1_′, P, F, A, L, W, or V; for P_2_′, L, Q, V, A, T, or I; for P_3_′, D, S, Q, T, M, V, R, or I; and for P_4_′, T, M, Q, V, P, G, R, or S. This resulted in a virtual library of 6 × 6 × 8 × 5 × 6 × 6 × 8 × 8 = 3,317,760 entries.

The library was first screened using the CLM to filter out all noncleavable substrates for the HXB2 HIV-1 protease. We considered a substrate noncleavable if its predicted cleavability parameter was less than −0.3. This resulted in 2,463,379 cleavable sequences (∼74% of the initial library). We then used the CRM to predict the actual rate of cleavage for the cleavable octapeptides. From these we chose 15 substrates; seven with a predicted cleavage rate log(k_cat_/K_m_) of more than 4.2 U, and eight with a rate less than 4.2 U. To ensure that peptides were dissimilar, we first randomly selected substrates with predicted log(k_cat_/K_m_) > 4.2 U, allowing at most four amino acids to be identical with the corresponding positions of the substrates in the dataset and in the already chosen substrates in the test set. If none of the remaining substrates met the requirements, five-amino-acid similarity was allowed. The same procedure was applied for the eight substrates with the predicted log(k_cat_/K_m_) < 4.2 U (Table 3, numbers 4–18). Next, we consecutively chose 15 substrates predicted to be noncleavable by the CLM by HXB2 HIV-1 protease, allowing at most four amino acids to be identical at any same positions among all the substrates already selected (including the cleavable substrates already chosen above). If none of the remaining substrates met the requirements, a five-amino-acid similarity was allowed (Table 3, numbers 19–33). We then used the CLM to predict cleavability of the chosen 30 substrates by mutant HIV-1 proteases I84V, L90M, and I84V + L90M. (The outcome for the predicted cleavability of the 30 chosen substrates was essentially the same for the mutant HIV-1 proteases as for the HXB2 HIV-1 protease.) Following this, we then again applied the CRM and predicted the cleavage rate of the 15 cleavable substrates chosen for the three mutant HIV-1 proteases, I84V, L90M, and I84V + L90M.

### Synthesis of novel retroviral protease substrates.

A total of 33 octapeptide sequences were engineered into internally quenched fluorogenic substrates. Fluorogenic substrates were synthesized by solid-phase peptide synthesis using an automated multiple peptide synthesizer (MultiPep; Intavis AG Bioanalytical Instruments, http://www.intavis.com; Table 3). Reagents were purchased from Fluka (http://www.fluka.org), Applied Biosystems (http://www.appliedbiosystems.com), Bachem (http://www.bachem.com), or Novabiochem (http://www.emdbiosciences.com/html/NBC/home.html). The following amino acid derivatives were used in the synthesis: Fmoc-L-Ala-OH, Fmoc-L-Arg(Pbf)-OH, Fmoc-L-Asn(Trt)-OH, Fmoc-L-Asp(O*t*-Bu)-OH, Fmoc-L-Gln(Trt)-OH, Fmoc-L-Glu(O*t*-Bu)-OH, Fmoc-L-Glu(EDANS)-OH, Fmoc-Gly-OH, Fmoc-L-Ile-OH, Fmoc-L-Leu-OH, Fmoc-L-Lys(Boc)-OH, Fmoc-L-Lys(DABCYL)-OH, Fmoc-L-Met-OH, Fmoc-L-Phe-OH, Fmoc-L-Pro-OH, Fmoc-L-Ser(*t*-Bu)-OH, Fmoc-L-Thr(*t-*Bu)-OH, Fmoc-L-Trp(Boc)-OH, Fmoc-L-Tyr(*t-*Bu)-OH; and Fmoc-L-Val-OH. PyBOP was used as an activating reagent and Tenta Gel R PHB-Arg(Pbf)-Fmoc resin (capacity 0.16 mmol/g) as a polymeric support.

The peptides were synthesized at a 5-μmol scale using the automated standard protocol optimized for Fmoc chemistry provided with the MultiPep synthesizer. Each cycle included deprotection of the Fmoc group by 20% piperidine in DMF and washing of the support with DMF; coupling (i.e., the N-deblocked peptidyl-resin was treated with a solution of the appropriate Fmoc amino acid derivative, PyBOP, and NMM in DMF for 25 min) and washing of the support with DMF, capping (i.e., treatment of the polymer with a 2% solution of acetic anhydride in DMF for 5 min), and washing of the support with DMF.

The final synthetic step on MultiPep included deprotection with 20% piperidine in DMF, washing of the support with DMF and CH_2_Cl_2_, and drying. The peptide was deprotected and cleaved from the resin with deprotection mixture (TFA–triisopropylsilane–1,2-ethanedithiol–water, 92.5:2.5:2.5:2.5) for 3 h at room temperature, triturated with *tert*-butyl-methyl ether, taken up in MeCN/water, lyophilized, and purified by high-performance liquid chromatography (HPLC); their structures were confirmed by mass spectrometry. Analytical HPLC was performed on a Waters (http://www.waters.com) system (Millenium32 workstation, 2690 separation module, 996 photodiode array detector) equipped with Vydac RP C18 90 Å reversed-phase column (2.1 × 250 mm; http://www.vydac.com).

Small-scale preparative HPLC was carried out on a system consisting of a 2150 HPLC pump, 2152 LC controller, and 2151 variable wavelength monitor (LKB, Sweden) and Vydac RP C_18_ column (10 mm × 250 mm, 90 Å, 201HS1010), with the eluent, an appropriate concentration of MeCN in water + 0.1% TFA, a flow rate of 5 mL/min, and detection at 280 nm. Freeze-drying was carried out at 0.01 bar on a Lyovac GT2 Freeze-Dryer (Steris Finn-Aqua**,** http://www.steris.com) equipped with a Trivac D4B (Leybold Vacuum, http://www.oerlikon.com) vacuum pump and a liquid nitrogen trap.

Peptides were checked by liquid chromatography/mass spectrometry using a Perkin Elmer PE SCIEX API 150EX instrument equipped with a turboionspray ion source (PerkinElmer Life and Analytical Sciences, http://las.perkinelmer.com) and a Dr. Maisch Reprosil-Pur C18-AQ (http://www.dr-maisch.com), 5 μm, 150 mm × 3 mm HPLC column, using a gradient formed from water and acetonitrile with 5 mM ammonium acetate additive.

When not otherwise specified, chemicals were reagent grade from Sigma (http://www.sigmaaldrich.com).

### Substrate cleavage assays.

Wild-type HIV-1 protease (HXB2 clone) and its three mutants, the I84V, L90M, and I84V + L90M genes, were a kind gift of Professor Helena Danielson, Uppsala University. Protease expression, isolation, refolding, and analysis were performed as previously described [72].

Rates of cleavage of the synthesized internally quenched fluorogenic substrates by the HXB2 and mutant HIV-1 proteases were assayed fluorimetrically (ex, 355 nm; em 490–10 nm) in black 96-well plates (Nunc, http://www.nuncbrand.com) under the conditions detailed in Table S3, assay 17, using a PolarSTAR OPTIMA microplate reader (BMG LABTECH, http://www.bmglabtech.com). Substrate stock solutions were 1 mM dissolved in DMSO (Table 3, numbers 1–33). A typical reaction mixture (total volume 100 μL) contained variable concentrations of peptide substrates in 0.1 M acetic acid and 1.1 M sodium chloride (pH 5.0 was achieved with sodium hydroxide solution) and 35 ng of enzyme. Reaction was conducted at 37 °C for 60 min (cycle time, 60 s, with 5 s shaking after each cycle). Each experiment was repeated at least three times, and the average value was taken as a final result (Table 3). The kinetic data was analyzed by nonlinear fit using the GraFit program and the basic equation for Michaelis–Menten kinetics [73]. The obtained k_cat_/K_m_ constants were converted into mM^−1^h^−1^ units for before further use.

## Supporting Information

Figure S1 Structural Alignment of Nine Wild-Type Retroviral Proteases (26 KB DOC)

Table S1 Summary of the Dataset for Retroviral Proteases Used Herein References to publications, description of the data considered under the study, and the number of entries each article added to the dataset are shown.
(38 KB DOC)

Table S2 The 61 Retroviral Proteases from Nine Retroviruses Included in the Study (21 KB DOC)

Table S3 Descriptors of Assay Conditions Used for Determination of Substrate Cleavage Rates (40 KB DOC)

### Accession Numbers

The Protein Data Bank (http://www.pdb.org) accession numbers for the proteins discussed in this paper are HIV-1 protease (1aid), HIV-2 protease (1ida), HTLV-1 protease (2b7f), FIV protease (4fiv), RSV protease (1bai), AMV protease (1mvp), and EIAV protease (1fmb). The Swiss-Prot (http://www.expasy.org/sprot) accession numbers for the proteases discussed in this paper are Mo-MuLV protease (P03355) and BLV protease (P10270).

## Abbreviations

AMV: avian myeloblastosis virus
BLV: bovine leukemia virus
CLM: cleavability model
CRM: cleavage rate model
EIAV: equine infectious anemia virus
FIV: feline immunodeficiency virus
HPLC: high-performance liquid chromatography
Mo-MuLV: Moloney murine leukemia virus, PLS, partial least squares
RMSECV: root mean square error of internal cross-validation
RMSEE: root mean square error of estimation
RMSEP: root mean square error of prediction
RSV: Rous sarcoma virus
STM: single target model
