# Supplementary material for: A Look Inside HIV Resistance through Retroviral Protease Interaction Maps
Source: PLoS Comput Biol. 2007 Mar 9;3(3):e48. doi: 10.1371/journal.pcbi.0030048 (PMC1817660; doi:10.1371/journal.pcbi.0030048)
Supplement: Supplementary file 1 [file pcbi.0030048.sg001.doc]

**Figure S1.** Structural alignment of nine wild-type retroviral proteases^*^. Amino acid residues highlighted in red are conserved among all proteases. Residues highlighted in blue cannot be structurally aligned between all proteases. Residues highlighted in yellow (85 amino acids) were the ones considered in the study.

```
                                10                  20        30              40          50
                                │                   │         │               │           │
HIV-1         -------PQITLW--QRPLVTIRIG----------GQLKEALLDTGADDTVLEE--M--N--LPGKWK--PKMIGGI
HIV-2         -------PQFSLW--KRPVVTAHIE----------GQPVEVLLDTGADDSIVAG--I--E--LGNNYS--PKIVGGI
RSV           -------LAMTMEHKDRPLVRVILTNTGSHPVKQRSVYITALLDSGADITIISEEDWP---TDWPVMEAANPQIHGI
AMV           -------LAMTMEHKDRPLVRVILTNTGSHPVKQRSVYITALLDSGADITIISEEDWP---ADWPVMEAANPQIHGI
FIV           --YNKVGTTTTLE--KRPEILIFVN----------GYPIKFLLDTGADITILNRRDFQ-VKN-SIENG--RQNMIGV
HTLV-1        -------PVIPLDPARRPVIKAQVDTQTSHP-----KTIEALLDTGADMTVLPIALFS----SNTPLK--NTSVLGA
Mo-MuLV       TLDDQGGQGQDPP--PEPRITLKVG----------GQPVTFLVDTGAQHSVLTQ--N--P--GPLSDK--SAWVQGA
BLV           -------LSIPLA-RSRPSVAVYLSGPWLQP---SQNQALMLVDTGAENTVLPQNWLV----RDYPRI--PAAVLGA
EIAV          -------VTYNLE--KRPTTIVLIN----------DTPLNVLLDTGADTSVLTTAHYNRLKYRGRKYQ--GTGIGGV

	                 60              70            80        90
	                 │               │             │         │
HIV-1         GGFIKVRQY--DQIPVEICG------HKAIGTVLVG----PTPVNIIGRNLLTQIGCTLNF------------------
HIV-2         GGFINTKEY--KNVEIEVLN------KKVRATIMTG----DTPINIFGRNILTALGMSLNL------------------
RSV           GGGIPMRKS-RDMIELGVINRDGSLERPLLLFPAVA----MVRGSILGRDCLQGLGLRLTNL-----------------
AMV           GGGIPMRKS-RDMIEVGVINRDGSLERPLLLFPAVA----MVRGSILGRDCLQGLGLRLTNL-----------------
FIV           GGGKRGTNY--INVHLEIRDENYKT-QCIFGNVCVL-EDNSLIQPLLGRDNMIKFNIRLVM------------------
HTLV-1        GGQTQDHFK-LTSLPVLIRLPFRT-TPIVLTSCLV---DTKNNWAIIGRDALQQCQGVLYLPEAKGPPVIL--------
Mo-MuLV       TGGKRYRWT--TDRKVHLAT------GKVTHSFLHV---PDCPYPLLGRDLLTKLKAQIHFEGSGAQVMGPMGQPLQVL
BLV           GGVSRNRYN-WLQGPLTLALKPEG-PFITIPKILV---DTFDKWQILGRDVLSRLQASISIPEEVRPPMVG--------
EIAV          GGNVETFST--P-VTIKKKG------RHIKTRMLVA----DIPVTILGRDILQDLGAKLVL------------------
```

^*^ Taken from: Bagossi P, Sperka T, Feher A, Kadas J, Zahuczky G et al. (2005) Amino acid preferences for a critical substrate binding subsite of retroviral proteases in type 1 cleavage sites. J. Virol. 79: 4213-4218.
