# Supplementary material for: A Look Inside HIV Resistance through Retroviral Protease Interaction Maps
Source: PLoS Comput Biol. 2007 Mar 9;3(3):e48. doi: 10.1371/journal.pcbi.0030048 (PMC1817660; doi:10.1371/journal.pcbi.0030048)
Supplement: Supplementary file 2 [file pcbi.0030048.st001.doc]

**Table S1.** Summary of the data set for retroviral proteases used herein. References to publications, description of the data considered under the study and the number of entries each article added to the data set are shown.

| **Reference** | **Description** | **Nr. of entries to the data set** |
| --- | --- | --- |
| [32] | Table 2. All experimentally determined k_cat_/K_m_ values Table 3. k_cat_/K_m_ values of P1F, P4’H, P1I and P4V substrates for AMV protease Table 3. All k_cat_/K_m_ values for RSV protease mutants R10K, L12V, I108V, I71V and I44V | 76 |
| [33] | Table I. All k_cat_/K_m_ values, except for P3F and P2H substituted substrates. Table II. All k_cat_/K_m_ values except for P3R, P3F, P1L, P1H, P1G and P1F substituted substrates | 57 |
| [34] | Table III. k_cat_/K_m_ values of P3R, P3F, P1L, P1H and P1G substituted substrates for AMV protease and RSV protease mutants (R105P+G106V and H65G+R105P+G106V). k_cat_/K_m_ values of P1W for RSV protease mutants | 20 |
| [35] | Table 1. All experimentally determined k_cat_/K_m_ values | 8 |
| [36] | Table V. k_cat_/K_m_ value of RSV NC-RT substrate for HIV-2 protease and k_cat_/K_m_ values of HIV-1 RT-IN substrate for AMV protease and RSV protease mutants (R105P+G106V; H65G+R105P+G106V) | 4 |
| [37] | Tables I-III. All experimentally determined k_cat_/K_m_ values | 44 |
| [38] | Table II. All experimentally determined k_cat_/K_m_ values | 22 |
| [39] | Table I. All k_cat_/K_m_ values except for MA-p2 and NC-PR substrates. Table II. All k_cat_/K_m_ values except for RT-IN substrate Table III. All k_cat_/K_m_ values except for MA-p2, NC-RT and PR-RT substrates. p2-p10 substrate is included Table VI. All k_cat_/K_m_ values except for PYVGSGLY(RR) substrate | 17 |
| [40] | Table I. k_cat_/K_m_ values for AMV protease and MA/p2A, p10/CA, CA/p3, p3/NC, PR/RT, MA/CA substrates. Non-cleavable substrates p10/CA and IN/p4 for HIV-1 protease. | 8 |
| [41] | Table 1. All k_cat_/K_m_ values. Table 2. k_cat_/K_m_ values for AMV protease and peptides Nr.10 and 12 | 5 |
| [42] | Table 1. All k_cat_/K_m_ values for BLV protease. k_cat_/K_m_ value for peptide in the “**^c^**” note | 7 |
| [43] | Table 1. k_cat_/K_m_ values for experiments Nr.2-12 (high ionic strength) and Nr.10-12 (low ionic strength) Table 2. k_cat_/K_m_ values for experiments Nr. 14-16, 18, 20, 21 Table 3. k_cat_/K_m_ value for HIV-2 protease and VSQNY*PEVQ substrate. Non-cleavable substrates SQNYPEVQ and SQNYPQVQ for MuLV and EIAV proteases Table 4. All k_cat_/K_m_ values except for HIV-1 protease | 34 |
| [44] | Table I. k_cat_/K_m_ value for experiment Nr.2 Table II. k_cat_/K_m_ values are calculated from k_cat_ and K_m_ values for EIAV protease and MA/CA, NC/p9, PR/RT. Non-cleavable substrate P15^pol^/IN is also considered for EIAV protease. Table III. All k_cat_/K_m_ values. (Calculated from the corresponding k_cat_ and K_m_ values) | 8 |
| [45] | Table 5. All k_cat_/K_m_ values except for HIV-1 protease | 8 |
| [46] | Table I. All k_cat_/K_m_ values except for HTLV-1 (wt) protease and CA/NC substrate experiment Table II. All k_cat_/K_m_ values | 14 |
| [47] | Table II. All k_cat_/K_m_ values from “Series A” group, except for P3Q substrates Table III. All k_cat_/K_m_ values for HIV-1 and HIV-2 proteases Table IV. All k_cat_/K_m_ values for HIV-1 and HIV-2 proteases Non-cleavable substrate with P2K for HIV-1 and HIV-2 proteases is included Table V. All k_cat_/K_m_ values for HIV-1 and HIV-2 proteases. Non-cleavable substrate with P2’K for HIV-1 and HIV-2 proteases is included Table VI. All k_cat_/K_m_ values for HIV-1 and HIV-2 proteases. Non-cleavable substrates with P1X (X=V, I, P, S, D, K or G) substitutions for HIV-1 and HIV-2 proteases are included | 67 |
| [48] | Table I. All k_cat_/K_m_ values except for Nr. 3 and 5 Table III. All k_cat_/K_m_ values except for Nr. 26 and 29 Table V. All k_cat_/K_m_ values except for Nr.17, 44 and 47. Nr.32 is included Table VI. All k_cat_/K_m_ values | 47 |
| [49] | Table I. All experiments included except “i”, “v” and “xi” for HIV-1 protease and “i” for HIV-2 protease Table II. All experiments included except “xiii” and “xx” for HIV-1 protease | 36 |
| [50] | Table I. Experiments included for HTLV-1 protease: Nr. 1-33, 35-46. Experiments included for HIV-1 protease: Nr. 11, 18-23, 27-33, 35-46, 49. k_cat_/K_m_ is considered 0.01 for HTLV-1 protease substrates Nr. 20 and 22. k_cat_/K_m_ is considered 100 for HIV-1 protease substrate Nr. 27 | 72 |
| [51] | Table 1. All k_cat_/K_m_ values | 27 |
| [52] | Table 1. All k_cat_/K_m_ values except for wild-type HIV-1 protease Table 2. k_cat_/K_m_ values calculated from the individual K_m_ and k_cat_ values under note “^a^” Table 3. All k_cat_/K_m_ values except for wild-type HIV-1 protease substrate Nr.1 Table 4. All k_cat_/K_m_ values except for wild-type HIV-1 protease substrate Nr.7 and for HIV-1 protease mutant (I84V) substrate Nr.9 | 88 |
| [53] | Table I. All k_cat_/K_m_ values except for wild-type HIV-1 protease CA-p2 and P6^pol^-PR substrates | 8 |
| [54] | Table II. k_cat_/K_m_ value for substrate SP-2014 Table III. All k_cat_/K_m_ values. (k_cat_/K_m_ =0.001 was set for substrates SP-2177 and SP-2179). 15 non-cleavable substrates described under “^c^” note are included | 59 |
| [55] | Table II. All k_cat_/K_m_ values except for “wild-type” and “NT” proteases Table III. All k_cat_/K_m_ values except for “wild-type” protease | 20 |
| [56] | Table 2. All k_cat_/K_m_ values except for “RSV GGGG-PR” protease substrate S_P1_Y | 10 |
| [57] | Table I. All k_cat_/K_m_ values for RSV (S9) protease | 5 |
| [58] | Table 2. All k_cat_/K_m_ values for HTLV-1 protease. k_cat_/K_m_ values for HIV-1 protease VSQVY*PIVQ and VSQIY*PIVQ substrates Table 3. All k_cat_/K_m_ values except for substrates KTKVL*VVQPK and KVL*VVQPK. All experiments marked “Not cleaved” are included as non-cleavable substrates | 77 |
| [59] | Table 2. Wild type HIV-1 protease non-cleavable substrates SQNYAIVQ, SQNYDIVQ and SQNYKIVQ are included. | 3 |
| [60] | Supplemental material table I. HIV-2 protease non-cleavable peptides ETTALVCD and AECFRIFD are included | 2 |
| [61] | Table 1. Four cleavage sites were observed cleaving fully denaturated human vimentin by HIV-1 protease. The non-cleavable octamers were extracted from five non-cleavable vimentin oligopeptides fragments between the cleavage sites by using an eight-residue-long sliding window. The first oligopeptide (52 amino acids) yielded in 45 non-cleavable octamers. The second fragment (9 amino acids) - 2 octamers, the third (32 amino acids) - 25 octamers, the forth (330 amino acids) - 323 octamers and the fifth (43 amino acids) - 36 octamers. (Sequence details were taken from <http://www.prospec.co.il/~prospec/cart/catalog/rHuVim.html>, Blast search: Homo sapiens: 7431, Vimentin) | 431 |
| [62] | Figure 1. Non-cleavable octamers were extracted from the RT sequence using an eight-residue-long sliding window. The following oligopeptides were considered: “-15...38” ***** (53 amino acids, 46 octamers), “39...87” (49 amino acids, 42 octamers), “88...115” (28 amino acids, 21 octamers), “116...231” (116 amino acids, 109 octamers), “232...303” (72 amino acids, 65 octamers), “304...325” (22 amino acids, 15 octamers), “326...436” (111 amino acids, 104 octamers), “441...483” (43 amino acids, 36 octamers), “484...494” (11 amino acids, 4 octamers), “495...532” (38 amino acids, 31 octamers), “533...560” (28 amino acids, 21 octamers) and a part of RNase H “PIHDHDHPFHGYQLEKEP” (18 amino acids, 11 octamers) **^*^** the numbers in brackets correspond to amino acid positions in RT sequence in figure 1 | 505 |
| [63] | Table II. All non-cleavable octamers | 239 |
| [64] | A denaturated hen egg lysozyme (129 amino acids, see [63]) is tolerant to HIV-2 protease cleavage. 122 non-cleavable octamers were extracted from the protein using an eight-residue-long sliding window | 122 |
| See Materials and Methods | Substrates Nr. 1-3 for HXB2 and 3 mutant (I84V, L90M and I84V+L90M) HIV-1 protease from Table 3 | 12 |
