# Supplementary material for: A Look Inside HIV Resistance through Retroviral Protease Interaction Maps
Source: PLoS Comput Biol. 2007 Mar 9;3(3):e48. doi: 10.1371/journal.pcbi.0030048 (PMC1817660; doi:10.1371/journal.pcbi.0030048)
Supplement: Supplementary file 3 [file pcbi.0030048.st002.doc]

**Table S2.** Summary of the sixty-one retroviral proteases from nine retroviruses included in the study.

| **Virus** | **Protease mutants** | **Number of protease variants** |
| --- | --- | --- |
| **HIV-1** | Wild type (HXB2); V32I^a^; V82T; I84V; R8K; L90M;G48V+L90M^b^; V82T+I84V; I84V+L90M; Q7K+L33I+L63I+C67A+C95A; 5Mut+R8Q^c^; 5Mut+D30N; 5Mut+K45I; 5Mut+M46L; 5Mut+G48V; 5Mut+V82S; 5Mut+V82A; 5Mut+N88D; 5Mut+L90M; 5Mut+K45I+L90M; 5Mut+K45I+V82S; 5Mut+D30N+V82S; 5Mut+N88D+L90M; 5Mut+K43E; 5Mut+I84V. | 25 |
| **HIV-2** | Wild type. | 1 |
| **RSV** | Wild type; R10K; L12V; I108V; I71V; I44V; R105P+G106V; H65G+R105P+G106V; R105P+G106V+S107N; S38T+V104T+S107N+M73V+A100L+R105P+G106V+I42D+I44V. | 10 |
| **AMV** | Wild type. | 1 |
| **FIV** | Wild type; I37V; I37+N55M; I37V+V59I; N55M; M56I; V59I; Q99V. | 8 |
| **HTLV-1** | Wild type; L40I; L40I+C90A+C109A. | 3 |
| **Mo-MuLV** | Wild type; E15R; H37D; V39I; W53I; V54I; V39I+V54I; A57I; V39I+A57I; Y63V; L92I. | 11 |
| **BLV** | Wild type. | 1 |
| **EIAV** | Wild type. | 1 |

# **Notes**

^a^ V32I denotes mutation at position 32 of HXB2 HIV-1 protease (substitution of Valine with Isoleucine).

^b^ Protease mutants with “+” sign indicate that there are two or more mutations within the protease sequence simultaneously.

^c^ “5Mut” denotes HXB2 HIV-1 protease with stabilizing Q7K+L33I+L63I+C67A+C95A mutations.
