# Supplementary material for: A Look Inside HIV Resistance through Retroviral Protease Interaction Maps
Source: PLoS Comput Biol. 2007 Mar 9;3(3):e48. doi: 10.1371/journal.pcbi.0030048 (PMC1817660; doi:10.1371/journal.pcbi.0030048)
Supplement: Supplementary file 4 [file pcbi.0030048.st003.doc]

**Table S3.** Descriptors of assay conditions used for determination of substrate cleavage rates. A1-A8 denote assay constituents as follows: A1, pH of assay; A2, sodium chloride in molar concentration; A3, 2-mercaptoethanol in mM concentration; A4, glycerol concentration (%); C5, dithiothreitol in mM concentration; C6, EDTA in mM concentration; A7, Nonidet-P40 concentration (%); C8, dimethylsulfoxide concentration (%).

| **Assay type** | **A1** | **A2** | **A3** | **A4** | **A5** | **A6** | **A7** | **A8** |
| --- | --- | --- | --- | --- | --- | --- | --- | --- |
| **1** | 5.9 | 1.0 | 0.2 | 0 | 0 | 0 | 0 | 0 |
| **2** | 5.9 | 1.0 | 0.2 | 0 | 0 | 0 | 0 | 4.0 |
| **3** | 5.9 | 0.8 | 0 | 0 | 0.2 | 0 | 0 | 0 |
| **4** | 5.6 | 2.0 | 0 | 7.5 | 5.0 | 1 | 0.2 | 0 |
| **5** | 5.6 | 2.0 | 0 | 5.0 | 5.0 | 1 | 0 | 0 |
| **6** | 5.6 | 2.0 | 0 | 7.5 | 0 | 1 | 0.2 | 0 |
| **7** | 5.6 | 2.0 | 0 | 7.5 | 2.5 | 1 | 0.1 | 0 |
| **8** | 5.6 | 0 | 0 | 7.5 | 2.5 | 1 | 0.1 | 0 |
| **9** | 5.6 | 2.0 | 0 | 5.0 | 7.5 | 1 | 0 | 0 |
| **10** | 5.6 | 2.0 | 0 | 7.5 | 5.0 | 1 | 0 | 0 |
| **11** | 5.9 | 2.4 | 0.2 | 0 | 0 | 0 | 0 | 4.0 |
| **12** | 5.9 | 2.4 | 0.2 | 0 | 0 | 0 | 0 | 0 |
| **13** | 5.9 | 2.4 | 0 | 0 | 0 | 0 | 0 | 0 |
| **14** | 5.9 | 2.4 | 0 | 0 | 0.2 | 0 | 0 | 0 |
| **15** | 6.0 | 3.0 | 0 | 0 | 0 | 0 | 0 | 0 |
| **16** | 5.25 | 0.2 | 0 | 5.0 | 1.0 | 0 | 0 | 5.0 |
| **17** | 5.0 | 1.0 | 0 | 0 | 0 | 0 | 0 | 1.6 |
